# Delayed CSF rhinorrhea presenting as a lethal acute bacterial meningitis 5 years post trauma

**DOI:** 10.1002/ccr3.7320

**Published:** 2023-05-09

**Authors:** Bipin Karki, Utsav Acharya, Bishika Pun, Pramesh Sunder Shrestha

**Affiliations:** ^1^ Department of Critical Care Medicine, Maharajgunj Medical Campus Institute of Medicine Kathmandu Nepal; ^2^ Department of Anaesthesia, Maharajgunj Medical Campus Institute of Medicine Kathmandu Nepal; ^3^ Department of Radiology Om Hospital and Research Center Kathmandu Nepal; ^4^ Maharajgunj Medical Campus Institute of Medicine Kathmandu Nepal

**Keywords:** CSF rhinorrhea, meningoencephalitis, septic shock

## Abstract

**Key clinical message:**

Delayed presentation of cerebrospinal fluid rhinorrhea is rare following head trauma. It is frequently complicated by meningitis if not addressed in time. This report highlights the importance of its timely management, the lack of which can lead to a fatal outcome.

**Abstract:**

A 33‐year‐old man presented with meningitis in septic shock. He had a history of severe traumatic brain injury 5 years back following which he had a history of intermittent nasal discharge for the past 1 year. On investigation, he was found to have *Streptococcus pneumoniae* meningitis, and CT scan of his head showed defects in the cribriform plate which established the diagnosis of meningoencephalitis secondary to cerebrospinal fluid rhinorrhea. The patient did not survive despite appropriate antibiotics.

## 1 INTRODUCTION

Cerebrospinal fluid (CSF) rhinorrhea may occur spontaneously, secondary to iatrogenic causes or post head trauma especially skull base fracture.^1^ The presentation is usually early, and delayed presentations after months or years are usually limited to case reports.^2^, ^3^, ^4^ In cases that present late, the incidence of occurrence of meningitis increases, which may lead to significant morbidity.^5^, ^6^ In this report, we discuss a case of a young man who developed intermittent CSF rhinorrhea 4 years after a head trauma. This was complicated by meningoencephalitis a year later, that is 5 years after head trauma which proved fatal to the patient.

## 2 CASE REPORT

A 33‐year‐old man presented with an acute onset of fever, headache, and vomiting for 2 days which was followed by restlessness for a day. The headache was more pronounced in the frontal region and was associated with multiple episodes of vomiting. There was no history of seizures or loss of consciousness. His past history was significant for a severe traumatic brain injury following a road traffic accident 5 years earlier for which he had been treated in an intensive care unit (ICU) for 9 days with mechanical ventilation for 4 days. The patient's family could not present any further details or provide previous medical records for review. Following this he had recovered well and was able to continue with his regular activities independently. However, he had a history of intermittent watery discharge from bilateral nostrils in the last 1 year. He had ignored it and did not seek any medical advice for the symptom. He did not have history of any surgical intervention or any other medical co‐morbidity.

**FIGURE 1:**
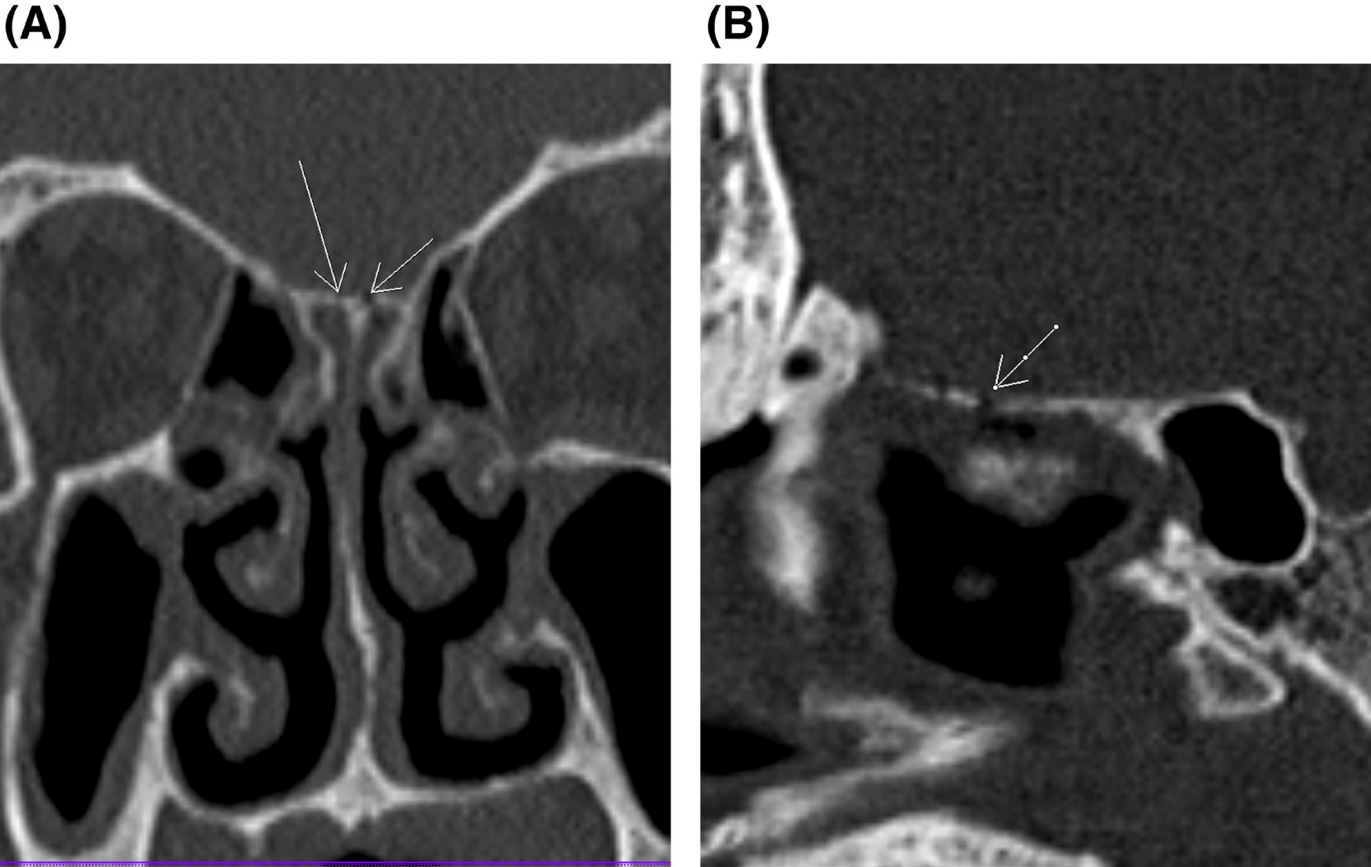
CT scan of the head with bone window. (A) Linear undisplaced fracture of cribriform plate and right lamella in coronal plane (arrows). (B) Fracture of cribriform plate in sagittal plane (arrow).

**FIGURE 2:**
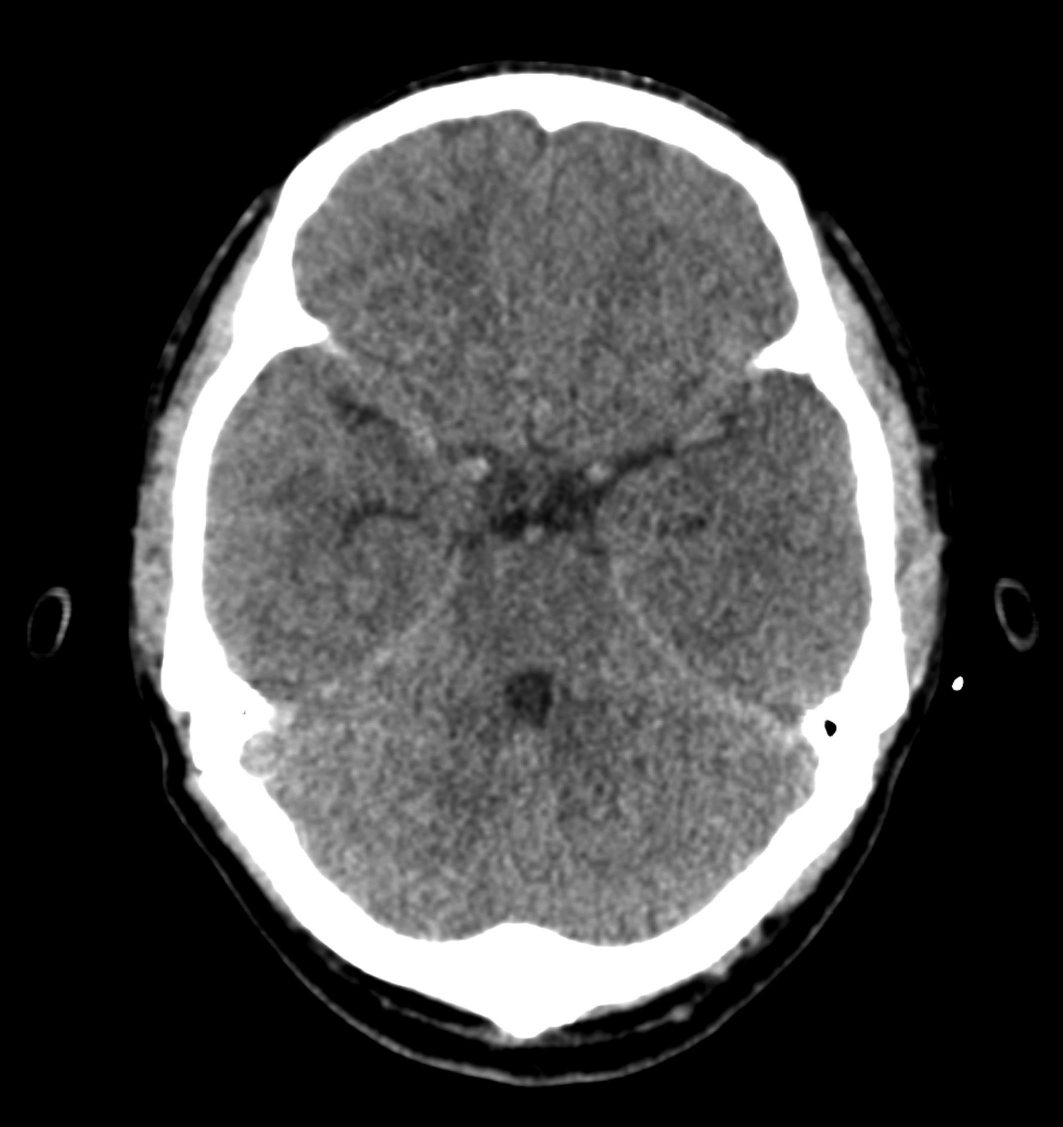
CT scan of the head showing cerebral edema.

On evaluation in the emergency department, he was restless. He had tachycardia with a heart rate of 130–140 beats per minute and tachypnea with a respiratory rate of more than 30 breaths per minute. His oxygen saturation was 89% at ambient air. His blood pressure was 70/30 mmHg measured in the brachial artery and his body temperature was 101 F. Oxygen supplementation and vasopressors were administered via infusion. His Glasgow Coma Scale (GCS) score was 6/15 (E1V1M4), and signs of meningismus were present. Pupils were bilaterally 2 mm in diameter, round, and reactive to light. Endotracheal intubation was done and mechanical ventilation was instated in view of low GCS for airway protection. An arterial blood gas was obtained which showed a high anion gap metabolic acidosis. Empiric antimicrobial therapy was started with intravenous vancomycin and meropenem for the provisional diagnosis of acute bacterial meningoencephalitis in septic shock. A computed tomography (CT) scan of the head was obtained which showed fracture of the cribriform plate (Figure 1A,B) with features of cerebral edema (Figure 2). Hyperosmolar therapy was commenced with 3% hypertonic saline and levetiracetam was also administered for seizure prophylaxis.

**FIGURE 3:**
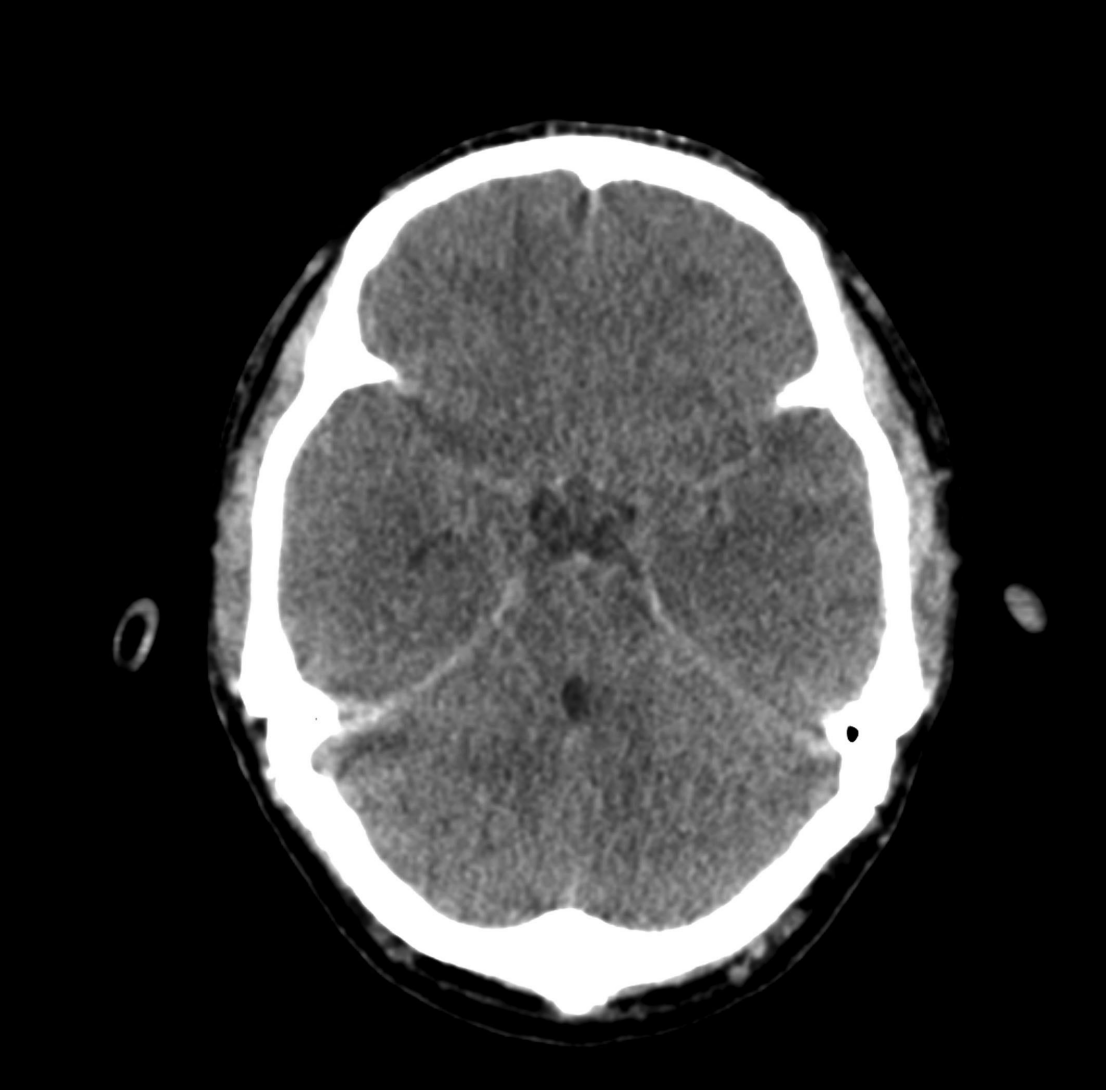
CT scan done 24 h later showing markedly increased cerebral edema with evidence of bilateral uncal herniation.

An electroencephalogram (EEG) was obtained in view of low GCS which showed encephalopathic changes. A diagnostic lumbar puncture was obtained which had a total leucocyte count of 350 cells/mm^3^ (85% neutrophils and 15% lymphocytes), sugar of 75 mg/dL and protein of 142 mg/dL. Within 12 h of presentation, the patient's pupils had become unequal with sluggish reaction to light. A repeat CT scan of the head showed increase in cerebral edema and features of uncal herniation (Figure 3). The patient succumbed to refractory shock on the second day of admission within 30 h of presentation at the hospital.

Microbial culture reports of the CSF was obtained after 48 h and showed growth of *Streptococcus pneumoniae*, sensitive to ceftriaxone and meropenem.

## 3 DISCUSSION

CSF rhinorrhea is not an uncommon complication of neurosurgical procedures or head trauma.^3^, ^4^ However, the presentation is usually early with more than 50% presenting within 48 h. Delayed presentations beyond a year are rare and are mostly limited to case reports.^7^ Later presentations are associated with higher complication rates, especially of meningitis which can lead to significant morbidity and as in our case mortality.^5^

Mechanisms for occurrence of delayed or occult leaks include (i) temporary closure of defect by herniation of brain matter in the foramina, (ii) resolution of edema later in the course of time, (iii) absorption of the hematoma plug that is initially sealing the foramen, (iv) contracture of scar tissue, or (v) tissue necrosis.^5^, ^7^ This may be missed many a time and often it is only after the development of complications like meningitis that CSF leaks are recognized.^3^

A single institution‐based review of 101 traumatic CSF leaks between 1984 and 1998 reported a 27.5% rate of occurrence of meningitis. Other complications included recurrent headache, pneumocephalus, and neurological deficits like hearing loss, which are usually sequelae of infection. The most common causative organism for meningitis isolated was *S. pneumoniae*. No mortalities were reported in this study.^2^ Though our patient was empirically started on antibiotics which were posthumously found to be effective for the isolated organism, the patient was unlucky to have succumbed probably due to the severity at presentation which included a high anion gap metabolic acidosis and shock.

In a study by Horst et al., CSF leak accounted for about 37% of community acquired bacterial meningitis with *S. pneumoniae* and *Haemophilus influenzae* being the most common pathogens responsible.^8^ Antibiotics have been recommended to confer protection against meningitis in cases with CSF rhinorrhea. However, the definitive management of CSF leak is surgical repair with antibiotic therapy considered only as prophylaxis until the defect is repaired.

## 4 CONCLUSION

Delayed presentation of posttraumatic CSF leak years after the initial trauma is very rare. Acute bacterial meningoencephalitis in such cases can prove fatal despite appropriate antibiotics.

## AUTHOR CONTRIBUTIONS

**Bipin Karki:** Conceptualization; methodology; writing – original draft; writing – review and editing. **Utsav Acharya:** Conceptualization; writing – original draft; writing – review and editing. **Bishika Pun:** Data curation; methodology; writing – original draft; writing – review and editing. **Pramesh Sunder Shrestha:** Methodology; supervision; writing – review and editing.

## FUNDING INFORMATION

No funding was obtained for this case report.

## CONFLICT OF INTEREST STATEMENT

The authors declare no conflict of interest.

## CONSENT

Written informed consent was obtained from the patient's wife for this case report.

## Data Availability

Not applicable since no new data were generated.

## References

[1] Li M , Mao S , Tang R , et al. Delayed diagnosis and treatment of cerebrospinal fluid leakage in current practice. J Craniofac Surg. 2019;30(6):1657‐1661. doi:10.1097/SCS.0000000000005402 30908435

[2] Friedman JA , Ebersold MJ , Quast LM . Post‐traumatic cerebrospinal fluid leakage. World J Surg. 2001;25(8):1062‐1066. doi:10.1007/s00268-001-0059-7 11571972

[3] Lee JJ , Kim HY , Dhong HJ , et al. Delayed cerebrospinal fluid leakage after treatment of skull base tumors: case series of 9 patients. World Neurosurg. 2019;132:e591‐e598. doi:10.1016/j.wneu.2019.08.067 31442635

[4] Chrysouli K , Papanikolaou V , Chrysovergis A , Kyrodimos E , Giotakis E . A clinical case of delayed posttraumatic frontal sinus cerebrospinal fluid leakage management via external surgical approach. J Craniofac Surg. 2022;33(7):2203‐2205. doi:10.1097/SCS.0000000000008493 36201687

[5] Poletti‐Muringaseril SC , Rufibach K , Ruef C , Holzmann D , Soyka MB . Low meningitis‐incidence in primary spontaneous compared to secondary cerebrospinal fluid rhinorrhoea. Rhin. 2012;50(1):73‐79. doi:10.4193/Rhino11.124 22469608

[6] Guyer RA , Turner JH . Delayed presentation of traumatic cerebrospinal fluid rhinorrhea Case report and literature review. Allergy Rhinol. 2015;6(3):188‐190. doi:10.2500/ar.2015.6.0132 PMC5391488 26686211

[7] Sharifi G , Mousavinejad S , Bahrami‐Motlagh H , et al. Delay posttraumatic paradoxical cerebrospinal fluid leak with recurrent meningitis. Asian J Neurosurg. 2019;14(03):964‐966. doi:10.4103/ajns.AJNS_95_18 31497141 PMC6703044

[8] Ter Horst L , Brouwer MC , van der Ende A , van de Beek D . Recurrent community‐acquired bacterial meningitis in adults. Clin Infect Dis. 2021;73(9):e2545‐e2551. doi:10.1093/cid/ciaa1623 33751028 PMC8563215

